# Nonocclusive mesenteric ischemia caused by type B aortic dissection: a case report

**DOI:** 10.1186/s12893-022-01656-2

**Published:** 2022-06-03

**Authors:** Mengchao Sheng, Wei Gong, Kui Zhao, Wei Li, Aimin Qian, Liuhui Chang, Yongyou Wu, Qiang Chen, Zhengrong Chen, Xiaodong Yang, Fengyun Zhong, Chungen Xing

**Affiliations:** Second Affiliated Hospital of Soochow University, Suzhou, China

**Keywords:** NOMI, Aortic dissection, Extensive resection, Segmental drainage, Case report

## Abstract

**Background:**

Nonocclusive mesenteric ischemia (NOMI) is defined as acute intestinal ischemia because of decreased blood flow in mesenteric vessels. Only a few cases of NOMI that occur secondary to aortic dissection (AD) have been reported, resulting in the lack of sufficient knowledge of diagnosis and treatment.

**Case presentation:**

We aimed to report a case of NOMI caused by type B Aortic Dissection. A 26-year-old male patient was transferred to our hospital with the diagnose of NOMI and aortic dissection in April 2018. The abdominal computed tomography (CT) assists the diagnosis of paralytic intestinal obstruction, intestinal wall pneumatosis, and perforation. Emergency laparotomy revealed that the bowel wall supplied by the superior mesenteric artery (SMA) was pale with the palpable but weak pulsation of the parietal artery. The small intestine was extremely dilated with a paper-thin, fragile wall that was ruptured easily and could not be sutured. In this case, extensive resection and segmental drainage were done. Postoperatively, the digestive tract was reconstructed. However, the patient suffered from iron deficiency anemia and short bowel syndrome eight months later, and unfortunately died from long-term complications.

**Conclusion:**

Aortic dissection leads to continuous decrease in blood pressure and blood flow to the SMA, considering as a predisposing factor for NOMI. During the treatment, extensive resection and segmental drainage are the optimal surgical strategy, which can make benefit in emergencies especially.

## Background

Nonocclusive mesenteric ischemia is a life-threatening disease that is defined as the ischemia and necrosis of intestinal tract without obstruction in mesenteric blood vessels [1], accounting for 20–30% of acute mesenteric ischemia with mortality of 30–60% [2, 3]. The concept of NOMI was first proposed by Ende in 1958 and speculated that heart failure was an essential condition for the onset of NOMI. At present, The mechanism is thought to be multifactorial, including large vessel vasoconstriction secondary to sympath-omimetics or vasoactive agents and hypoperfusion of mesenteric vessels from anemia, hypoxia, cardiovascularshock, and sepsis [4]. In contrast to vascular occlusion, the symptoms of NOMI are atypical at the early stage, which often leads to delayed diagnosis and high death rate due to serious underlying diseases [5]. Therefore, NOMI is relatively common in intensive care medicine, with the incidence of 0.2–0.3% [6]. Because of the extensive decreasing blood supply to the wall of the small intestine or colon, most parts of the bowel need to be resected, and postoperative short bowel syndrome is inevitable. Here, we report a rare case of NOMI secondary to type B aortic dissection.

## Case presentation

**Fig. 1:**
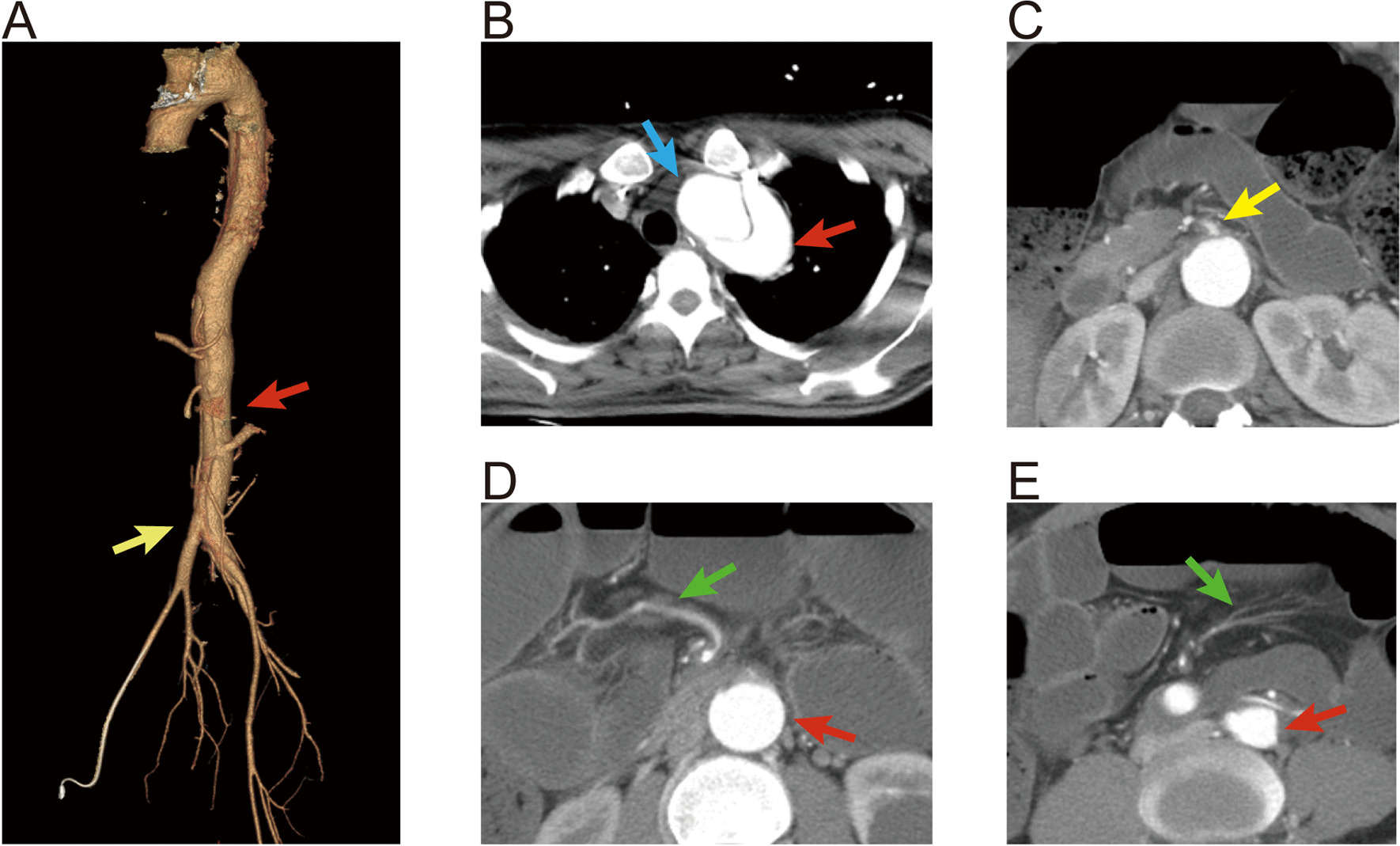
Thoracic and abdominal CTA of the patient showed type B aortic dissection (the true lumen and the false lumen of abdominal aorta were respectively marked with red and blue arrows, **B**) with a coarctated lumen of the initial segment of the superior mesenteric artery (yellow arrows, **A** and **C**) and a noticeable vessel tree in the enhanced sequence (green arrows, **D** and **E**)

**Fig. 2:**
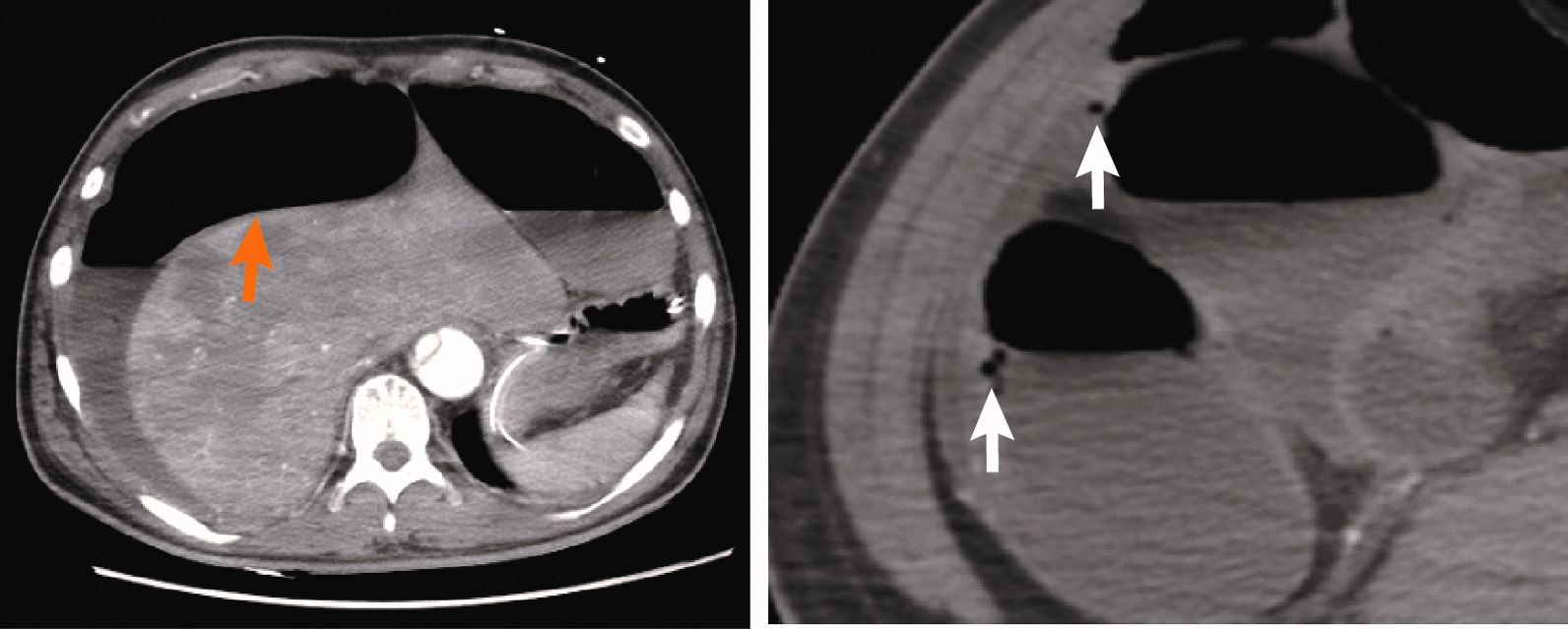
Abdominal computed tomography of the patient revealed a distended bowel with a paper-like thin wall (orange arrows) and gas accumulation (white arrows) in the small intestine wall

A 26-year-old male patient felt chest and back tearing pain and developed a coma four days later. He was diagnosed with subarachnoid hemorrhage and hydrocephalus in another hospital. External brain drainage was undergone immediately, and his consciousness recovered after the operation. Computed tomography angiography (CTA) showed aortic dissection with intracranial aneurysms of the left vertebral artery and basilar artery. Then the patient was transferred to our hospital. Seven days later after admission, his condition suddenly deteriorated with shock (BP 85/58 mmHg) and hypoxemia (PaO_2_ 58 mmHg) accompanied by shortness of breath, nausea, vomiting, and abdominal distension, but without abdominal pain or early signs of peritonitis. Thoracic and abdominal CTA showed type B aortic dissection with the coarctated lumen of the initial segment of the superior mesenteric artery and a noticeable vessel tree in the enhanced sequence (Fig. 1). The small intestine dilated and presented signs of paralytic intestinal obstruction. An ileus tube was inserted but the abdominal distension was not relieved significantly, and the symptoms of peritonitis gradually appeared. Abdominal CT showed that the small intestine was still extremely dilated with a paper-like thin wall even though the ileus tube was in the correct position. Intestinal wall pneumatosis was also observed (Fig. 2). There was a sizeable gas–liquid level in the right subphrenic region which led to the diagnosis of perforation and emergency laparotomy.

**Fig. 3:**
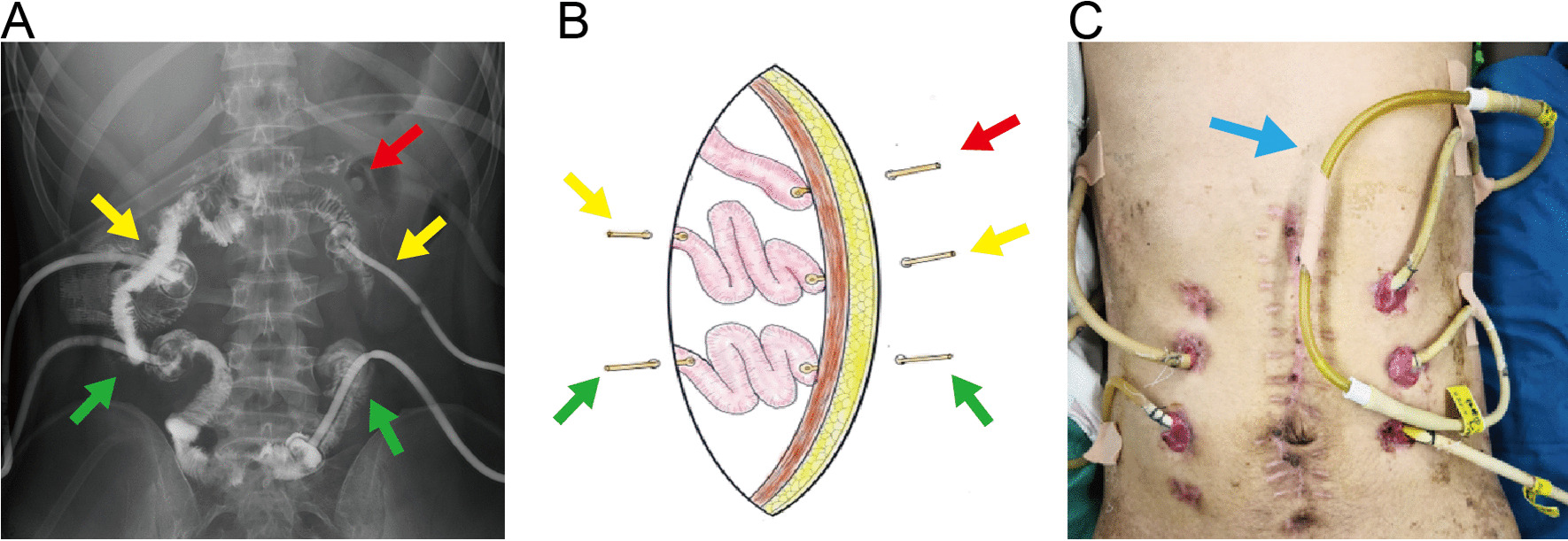
The CT image (**A**), graphical representation (**B**) explaining the location of the reserved small intestine marked as No. 1 (red arrows), No. 2 (yellow arrows) and No. 3 (green arrows). The photo (**C**) shows that the NO. 1 and NO. 2 intestines were bridged by rubber tubes(blue arrows). The intestinal movement direction was from left to right

At operation, the bowel wall supplied by the superior mesenteric artery appeared pale with palpable but weak pulsation of the peripheral artery, though there is no purplish-black necrosis. The small intestine was unable to be sutured since it was extremely dilated and easily to be ruptured due to a paper-thin and fragile wall. Besides, there were two typical perforated holes in the cecum and hepatic flexure of the colon with a significant amount of suppurative fluid in the abdominal cavity. Most of the small intestine and the whole right hemicolon were resected. The initial part of the jejunum approximately 8 cm long and the other two segments of the intestine approximately 55 and 37 cm long remained and were marked as No. 1, No. 2, and No. 3. Thickness of intestinal wall was the leading indicator for preservation. Pezzer's catheter was placed at the end of each segment and drawn out through the abdominal wall, and the end of the small intestine was fixed on the peritoneum (Fig. 3).

The postoperative course was complicated. When radiography revealed that there is no leakage from the remaining intestinal segments, enteral nutrition started to be given gradually, which is beneficial for intestinal resuscitation and early nutritional support.

**Fig. 4:**
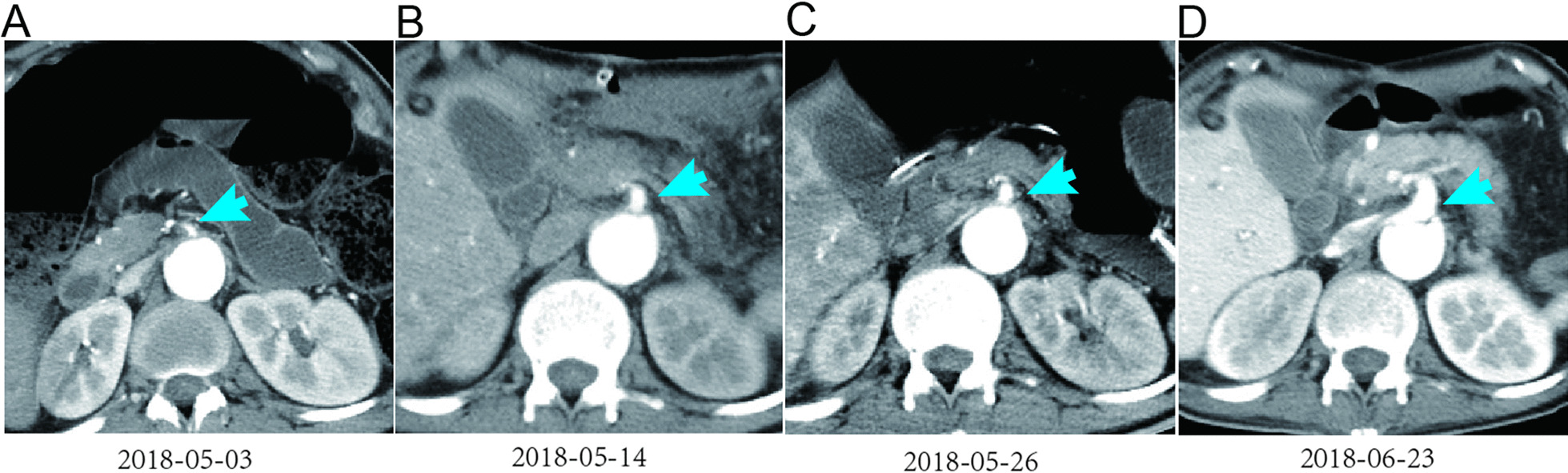
Abdominal computed tomography of the patient with aortic dissection after the intracavitary aortic repair **D** showed that the diameter of the SMA increased significantly compared to that before the operation (**A**–**C**)

On June 19, the patient underwent endovascular repair of the aorta. Stents were placed in the left common carotid artery, innominate artery, and thoracic aorta. Postoperative angiography showed that the diameter of the true lumen of the abdominal aorta was widened, and the diameter of the SMA was also significantly widened (Fig. 4). The blood supply to the digestive tract was improved.

Four days later, blood clots gushed from the NO. 3 intestine. We used a choledochoscope to detect the cause of bleeding because it was thin enough to pass through the drainage. A large clot was found in the middle part of the small intestine, tightly adhered to the intestinal wall with no active bleeding. Reperfusion injury was speculated to be the main reason for the bleeding after aortic repair. From July 11 to August 20, the NO. 1 and NO. 2 intestines were reconnected. The patients could intermittently take liquid food and enteral nutrition orally, while the supplementation of parenteral nutrition solution was reduced. Because of the obstruction, the NO. 3 intestine was not bridged to other segments.

Reconstruction of the digestive tract was performed on August 22. At operation, it was found that the middle part of the NO. 3 small intestine adhered tightly to the abdominal wall of the right iliac fossa and formed an angle. The digestive tract was reconstructed with end-to-end anastomosis, and the NO. 3 small intestine was anastomosed in the reverse peristaltic direction. The remaining small intestine was approximately 100 cm long. Postoperatively, the patient began to eat food on the seventh day and made a gradual recovery.

The patient ate regular food and did not need parenteral nutrition during the first eight months of follow-up. He maintained a weight above 49 kg, hemoglobin at a level of 110 g/L, and serum albumin greater than 40 g/L. He was also able to carry out light physical labor. However, after March 2019, his hemoglobin decreased progressively, and stool occult blood was positive. The test found that serum ferritin and vitamin B12 were low. Digestive endoscopy revealed multiple small polyps in the residual intestine. The patient was diagnosed with iron deficiency anemia, and it was challenging to rectify this condition by giving vitamin B12, folic acid, and iron. Therefore, blood transfusion and parenteral nutrition were needed intermittently. Unfortunately, the patient's condition worsened in the following days. With chronic heart failure and malnutrition appearing, ectopic bacteria from the digestive tract, sepsis, and uncontrolled toxic shock finally occurred. The patient died in August 2021.

## Discussion and conclusions

NOMI is associated with vasoconstriction of the mesenteric arteries, which causes intestinal hypoperfusion, and the blood flow redistributes to vital organs in the case of circulatory system failure [7]. The causes of NOMI include older age, cardiovascular disease, cardiac surgery, usage of vasoactive drugs (digitalis, ergotamine), shock, sepsis, dialysis, and hypotension after abdominal surgery [8, 9]. Reports [1, 3] based on the data from the International Registry of Acute Aortic Dissection showed that 71 cases (7.6%) were complicated with mesenteric ischemia, and 26 cases died (36.6%) out of 1034 cases of type B aortic dissection from 1996 to 2013. Although there was no clear distinction between occlusive and nonocclusive patients in this paper, it was confirmed by other case reports that aortic dissection could cause NOMI [10].

The clinical manifestations vary individually, depending on the degree of the narrowed vascular lumen [11]. However, the diagnosis of NOMI at an early stage is still difficult because of the atypical clinical symptoms and the lack of meaningful laboratory indicators. In this case, the patient did not undergo laparotomy initially because he did not have any signs of peritonitis, which may lead to delays in treatment. Another reason is that NOMI is always covered by other severe underlying diseases, such as hypovolemic shock, sepsis, and heart surgery.

Although there is currently still no standard treatment strategy for NOMI because of the variable etiologies, the consensus is that restoring adequate blood flow to the vessels at the early stage is critically important. Intraarterial infusion of vasodilators is an effective and safe treatment strategy, which can prevent intestinal necrosis and reduce mortality significantly [12, 13]. In contrast to other cases, the mechanism of small intestine ischemia caused by aortic dissection lead to continuous decrease in blood pressure and blood flow to the SMA. In this case, the diameter of SMA was widened significantly after the operation of endovascular repair, which shows that prompt treatment of aortic dissection can contribute to the improvement of prognosis.

Emergency laparotomy is necessary if intestinal wall necrosis is suspected, and enterectomy can effectively improve the survival rate [14]. In severe cases, intestinal ischemia is diffuse with no clear border for resection, the paper-thin wall is ruptured so easily that it cannot be sutured, and the extensive resection is performed, as seen in this paper. Thickness of the bowel wall is the most critical indicator for preservation, although the intestine is not good enough. From the analysis of 30 NOMI patients, Nakamura F found that 33.3% (7/21) of patients requiring additional intestinal resection in the planned second-look operation, because of progressed ischemia or necrosis 24–48 h after the first operation [15]. We did not reconstruct the alimentary tract immediately, and the preserved intestines were drained separately. When primary disease has not been corrected or the situation is severe, segmented drainage can avoid the impact of anastomotic leakage, progressed intestinal necrosis, perforation and another emergency operation, which is beneficial for the patient to pass through the difficult period.

The length of the small intestine is an essential index of intestinal digestion and absorption capacity. Segmental drainage can preserve the intestinal tract as much as possible, especially when the blood supply of intestinal wall is not enough. When the segments were confirmed to be in good condition, the residual bowel could be reconnected flexibly by tubes in vitro. In this way, patients can receive enteral nutrition early to maintain intestinal function, reduce liver injury and decrease parenteral nutrition. In addition, it is also good for the treatment of abnormal conditions in a minimal way, such as bleeding and obstruction.

To delay the passage of intestinal contents and increase the absorption of nutrients, Intestinal NO. 3 was anastomosed in the reverse peristaltic direction. The leading nutritional indicators were stable for the first eight months. Unfortunately, short bowel syndrome inevitably emerges, which in turn illustrates the importance of preserving the small intestine as much as possible.

In conclusion, NOMI caused by aortic dissection is so rare with the lack of sufficient knowledge. Immediate treatment should be taken when the bowel is extremely dilated, the paralytic intestinal obstruction cannot be alleviated by ileus tube, and imaging manifestations are characteristic. The bowel should be preserved as much as possible to prevent long-term short bowel syndrome. In cases of intestinal necrosis and secondary perforation that requires emergent surgery, extensive resection and segmental drainage are the optimal surgical strategy.

## Data Availability

The patient data and clinical images adopted are contained in the medical files of Second Affiliated Hospital of Soochow University, Jiangsu, China.

## Abbreviations

NOMI: Nonocclusive mesenteric ischemia
AD: Aortic dissection
SMA: Superior mesenteric artery
CT: Computed tomography
CTA: Computed tomography angiography
MDCT: Multidetector computed tomography
